# Multimorbidity and social determinants of health in the US prior to the COVID-19 pandemic and implications for health outcomes: a cross-sectional analysis based on NHANES 2017–2018

**DOI:** 10.1186/s12889-023-15768-8

**Published:** 2023-05-15

**Authors:** Bijan Mossadeghi, Roberta Caixeta, Dolores Ondarsuhu, Silvana Luciani, Ian R. Hambleton, Anselm J. M. Hennis

**Affiliations:** 1 Stony Brook University Medical Center, 101 Nicolls Road Health Sciences Center, Stony Brook, NY 11794-843 USA; 2 New York, United States; 3 Pan American Health Organization, Washington, DC USA; 4 Washington D.C., United States; 5 George Alleyne Chronic Disease Research Centre, The University of the West Indies, Bridgetown, Barbados; 6 Bridgetown, Barbados

**Keywords:** Multimorbidity, Social determinants of health, NHANES, COVID-19

## Abstract

Multimorbidity increases the risk of all-cause mortality, and along with age, is an independent risk factor for severe disease and mortality from COVID-19. Inequities in the social determinants of health contributed to increased mortality from COVID-19 among disadvantaged populations. This study aimed to evaluate the prevalence of multimorbid conditions and associations with the social determinants of health in the US prior to the pandemic.

**Methods** Data from the 2017–18 cycle of NHANES were used to determine the prevalence of 13 chronic conditions, and the prevalence of having 0, 1, or 2 or more of those conditions, among the US adult population aged ≥ 20 years. Multimorbidity was defined as having 2 or more of these conditions. Data were stratified according to demographic, socioeconomic and indicators of health access, and analyses including logistic regression, performed to determine the factors associated with multimorbidity.

**Results** The prevalence of multimorbidity was 58.4% (95% CI 55.2 to 61.7). Multimorbidity was strongly associated with age and was highly prevalent among those aged 20–29 years at 22.2% (95% CI 16.9 to 27.6) and continued to increase with older age. The prevalence of multimorbidity was highest in those defined as Other or multiple races (66.9%), followed in decreasing frequency by rates among non-Hispanic Whites (61.2%), non-Hispanic Blacks (57.4%), Hispanic (52.0%) and Asian (41.3%) groups.

Logistic regression showed a statistically significant relationship between multimorbidity and age, as expected. Asian race was associated with a reduced likelihood of 2 or more chronic conditions (OR 0.4; 95% CI 0.35 to 0.57; *P* < 0.0001). Socioeconomic factors were related to multimorbidity. Being above the poverty level (OR 0.64; 95% CI 0.46 to 0.91, *p* = 0.013); and a lack of regular access to health care (OR 0.61 (95% CI 0.42 to 0.88, *p* = 0.008) were both associated with a reduced likelihood of multimorbidity. Furthermore, there was a borderline association between not having health insurance and reduced likelihood of multimorbidity (OR 0.63; 95% CI 0.40 to 1.0; *p* = 0.053).

**Conclusions** There are high levels of multimorbidity in the US adult population, evident from young adulthood and increasing with age. Cardiometabolic causes of multimorbidity were highly prevalent, especially obesity, hyperlipidemia, hypertension, and diabetes; conditions subsequently found to be associated with severe disease and death from COVID-19. A lack of access to care was paradoxically associated with reduced likelihood of comorbidity, likely linked to underdiagnosis of chronic conditions. Obesity, poverty, and lack of access to healthcare are factors related to multimorbidity and were also relevant to the health impact of the COVID-19 pandemic, that must be addressed through comprehensive social and public policy measures. More research is needed on the etiology and determinants of multimorbidity, on those affected, patterns of co-morbidity, and implications for individual health and impact on health systems and society to promote optimal outcomes. Comprehensive public health policies are needed to tackle multimorbidity and reduce disparities in the social determinants of health, as well as to provide universal access to healthcare.

## Background

Multimorbidity, defined by the World Health Organization as the co-occurrence of two or more chronic medical conditions in one person [1], increases with older age and is associated with more complex care, reduced quality of life and higher mortality [2], constituting one of the greatest challenges facing health systems presently and into the foreseeable future [3].

Multimorbidity is more frequent in disadvantaged groups and is strongly linked to inequities in the social determinants of health [1–4]. Increased mortality from COVID-19 has been evident among older groups with high rates of chronic diseases, as well as disadvantaged populations [5, 6].

The US has had the highest mortality rate from COVID-19 among the industrialized nations [7]. There are documented associations between multimorbidity and socioeconomic factors and given the higher COVID-19 related mortality in groups living in vulnerable situations, we aimed to describe the frequency and distribution of multimorbid conditions and evaluate associations between multimorbidity and the social determinants of health in the US adult population in the immediate pre-pandemic period. This information is expected to contribute to knowledge of factors related to high rates of mortality during the pandemic and provide evidence to inform public policies and research to strengthen health systems, as well as preparedness and responses to health emergencies.

## Methods

### Study population

This study is based on the 2017–18 cycle of The National Health and Nutrition Examination Survey (NHANES), the most recent period of data collection prior to the COVID-19 pandemic. NHANES uses a complex, multistage, probability design resulting in the study sample being representative of the civilian noninstitutionalized population, and includes demographic, socioeconomic, dietary, and health information, gathered via interviews with participants. A subset of participants undergoes more detailed laboratory examinations. Written informed consent is obtained from all participants. Survey protocols and Institutional Review Board approvals are available on the NHANES website [8].

### Study participants

In 2017–2018, 16,211 persons were selected for the NHANES survey and 9254 completed the interview. Since the incidence of cardiovascular disease is extremely low at younger ages, the present study was limited to adult participants aged 20 years and older. This group comprised a total of 5569 participants (representative of the more than 238 million adults in the United States) who were interviewed, and 5265 who also underwent physical and laboratory examinations.

### Multimorbidity

Multimorbidity was defined as the presence of 2 or more chronic conditions in an individual and chronic conditions defined as cardiovascular disease, heart failure, chronic kidney disease, asthma, chronic obstructive pulmonary disease, arthritis, cancer, stroke, hypertension, hyperlipidemia, diabetes, and obesity, consistent with previous evaluations [9]. Liver disease was also included in this evaluation because of its strong association with lifestyle factors such as alcohol consumption. Multimorbidity was categorized as 0 (no morbidities), 1 morbidity, or 2 or more morbidities.

Most of the conditions evaluated were based on participant self-report. The survey questions “have you ever been told by a doctor that you have *a specific medical condition*?” were used to classify participants as having congestive heart failure, asthma, chronic obstructive pulmonary disease, arthritis, stroke, cancer, and liver disease, if they gave a positive response. Similarly, participants were classified as having cardiovascular disease if they responded affirmatively to being told that they had coronary heart disease or had a heart attack. Chronic kidney disease (CKD) was defined as giving a positive response to being told that they had weak or failing kidneys.

Participants were classified as having hypertension based on measured systolic blood pressure ≥ 140 or diastolic blood pressure ≥ 90 mmHg [10] (calculated as the average of the 2nd and 3rd measurements, or as the 4th measurement if one existed), or if they had been told they had high blood pressure and were taking an anti-hypertensive medication. Participants were classified as having hyperlipidemia and diabetes if they gave a positive answer to the self-reported question or had a lab value equal to or greater than 200 mg/dL for total cholesterol [11] or 6.5% for HgbA1c, respectivley [12]. Obesity was defined as a body mass index ≥ 30 kg/m^2^ [13].

### Covariates

Demographic characteristics evaluated were consistent with previous studies on multimorbidity [4], and included age, sex, race, and socioeconomic status (education level, household income, ratio of family income to poverty level), health insurance status, and access to healthcare. Age was divided into 10-year strata starting from 20 years. Race was characterized as Hispanic, non-Hispanic White, non-Hispanic Black, Asian, and mixed-race (defined by NHANES as including Native-American, Hawaiian, and Pacific Islander). Participants’ education level was categorized as “not high school graduate” and “high school and above.” Annual household income was categorized as “below $20,000” and “above $20,000”, and the ratio of family income to poverty was recoded as “above poverty” for greater than 1.0 and “at or below poverty” for less than or equal to 1.0. The health insurance status of participants was defined as “yes” or “no” for respectively, having or not health insurance. Access to healthcare was defined as responding “yes” to having a regular place for healthcare, or that “there is no place”, or “there is more than one place”.

### Statistical analysis

All data analyses were performed with SAS Studio version 9.4 (SAS institute Inc., Cary, NC). Appropriate sampling weights and SAS survey analysis procedures were used according to NHANES analytic and reporting guidelines to account for the complex survey design, oversampling of ethnic minority groups, survey nonresponse, and poststratification. Missing data were assumed to be missing at random, and Taylor Series Linearization was used to calculate variances. Responses of “refused” or “don’t know” were included in the denominator for calculation of prevalence but were excluded for calculation of Chi Squared *p* values, since the prevalence values of 0 in some instances precluded the use of the test. Chronic diseases prevalence was stratified by age, sex, race, education level, poverty level, income, health insurance status, and access to a regular place for healthcare. Rao-Scott Chi-Squared and logistic regression analyses were calculated by using SAS survey analysis procedures. Statistical significance was determined based on a *P* value < 0.05, and 95% confidence intervals are provided at all times for a more complete picture of estimation uncertainty.

Additionally, we conducted sensitivity analyses of the effect of specific variables, namely income and education on multimorbidity outcomes using alternative cutpoints for stratification, as well as sensitivity analysis of our multimorbidity regression model to assess the contribution of each of the 13 component conditions.

## Results

The prevalence of cardiometabolic conditions among adults aged 20 years and older is presented in Table 1. The overall prevalence of hypertension was 33.1% (95% CI 30.0–36.3). An association was apparent between age and prevalent hypertension, which increased linearly from 2.7% among persons aged 20 to 29 years, to 72% among those in their 80 s, with a slightly higher prevalence among older women. Among racial/ethnic groups the highest prevalence of hypertension was found among non-Hispanic Blacks (42%), intermediate (30–33%) among Whites, Asians, and mixed races/Native-Americans, and lowest among Hispanics (25%). The overall prevalence of hyperlipidemia was 53.0% (95% CI 49.5–56.4%), diabetes 13.6% (95% CI 12.4–14.8%), and obesity 42.3% (95% CI 38.7–45.8%). Non-Hispanic Blacks had the lowest overall prevalence of hyperlipidemia at 43.8% (95% CI: 40.4–47.2); rates of diabetes were comparable across ethnic groups (*P* = 0.02), while Asians had considerably lower rates of obesity 17.3% (95% CI: 15.0–20.0 Based on a lower cut-point of 27.5 kg/m^2^ for obesity in the Asian subgroup [14], the difference in the risk of multimorbidity between Asian and non-Hispanic White participants was less pronounced, with the associated odds ratio rising from 0.44 (95% CI 0.35 to 0.57) to 0.61 (95% CI 0.44 to 0.85). However, given the lower precision of this estimate and similar effect measure, we conducted further analyses using the BMI cutpoint of 30 kg/m^2^.

**Table 1:** Prevalence of specific chronic health conditions by sociodemographic characteristics in US adults: NHANES 2017–18

Sociodemographic characteristics	**Hypertension**	**Hyperlipidemia**	**Diabetes**	**Obesity**
	% (95% CI)	% (95% CI)	% (95% CI)	% (95% CI)
**Overall**	33.1 (30.0—36.3)	53.0 (49.5—56.4)	13.6 (12.4—14.8)	42.3 (38.7—45.8)
*Sex*				
Male	34.3 (30.4—38.3)	52.8 (48.9- 56.8)	14.4 (12.5—16.3)	42.4 (36.9 – 48.0)
Female	32.0 (29.0—35.1)	53.1 (48.7—57.4)	12.8 (10.5—15.2)	41.9 (38.0—45.8)
Rao-Scott Chi-Square Test *P* value	0.13	0.93	0.43	0.75
*Age Group*				
20–29	2.7 (1.4—4.0)	22.0 (16.9—27.2)	1.8 (0.6—3.0)	36.8 (28.6—44.9)
30–39	14.4 (10.8—18.1)	39.3 (34.8—43.8)	5.0 (3.0—6.9)	43.5 (38.5—48.4)
40–49	21.6 (17.0—26.2)	52.4 (46.3—58.6)	10.3 (6.4—14.1)	44.9 (39.7—50.2)
50–59	45.1 (36.4—53.8)	66.5 (59.8—73.1)	16.9 (12.2—21.6)	44.5 (39.5—49.4)
60–69	56.4 (50.1—62.7)	72.7 (67.9—77.5)	22.5 (18.0—27.1)	44.3 (36.9—51.7)
70–79	68.3 (63.4—73.1)	74.7 (70.4 – 79.0)	31.0 (26.8- 35.2)	43.2 (35.8—50.5)
80–89	72.7 (68.2—77.1)	67.3 (63.1—71.6)	28.2 (23.6—32.8)	30.5 (25.9—35.1)
Rao-Scott Chi-Square Test *P* value	** < 0.0001**	** < 0.0001**	** < 0.0001**	**0.02**
*Race*				
Hispanic	25.2 (21.1—29.4)	50.7 (45.3—56.0)	13.6 (11.4—15.8)	44.2 (41.0—47.4)
Non-Hispanic White	33.9 (29.5—38.2)	55.2 (50.6—59.8)	12.8 (11.0—14.7)	42.5 (37.5—47.5)
Non-Hispanic Black	42.4 (38.2—46.6)	43.8 (40.4—47.2)	15.4 (12.8—18.1)	48.6 (45.6—51.6)
Asian	30.0 (26.8—33.3)	50.3 (45.0—55.7)	16.0 (13.8—18.3)	17.3 (15.0—19.7)
Other or multiple races	31.8 (22.7—40.9)	56.8 (46.8—66.8)	16.4 (11.8—21.0)	46.1 (34.63- 57.6)
Rao-Scott Chi-Square Test *P* value	** < 0.0001**	**0.0009**	**0.02**	** < 0.0001**

The overall prevalence of multimorbidity among US adults is 58.4% (95% CI 55.2–61.7), with similar distributions among men and women (Table 2). The prevalence of adults with multimorbidity increased with older age, peaking in the 70–79 age group. Of note, multimorbidity is highly prevalent even at younger ages (22% in those aged 20–29 years). Rates of 2 or more conditions were highest in those of Other or multiple races (66.9%), followed in decreasing frequency by rates among non-Hispanic Whites (61.2%), non-Hispanic Blacks (57.4%), Hispanic (52.0%) and Asian (41.3%) groups. These differences, however, were not statistically significant. Socioeconomic factors such as education or income were not related to multimorbidity, while in contrast, having health insurance or a regular place for healthcare were strongly associated with having 2 or more chronic conditions (*P* < 0.0001 in both scenarios).

**Table 2:** Distribution of US adults with chronic conditions by sociodemographic characteristics: NHANES 2017–18

Sociodemographic characteristics	**No conditions**	**1 condition**	**2 or more conditions**	**Chi-Square Test**
	% (95% CI)	% (95% CI)	% (95% CI)	*P* value
**Overall**	17.3 (14.6—19.9)	24.3 (22.6—25.9)	58.4 (55.20- 61.7)	
*Sex*				
Male	16.9 (13.9—19.9)	24.4 (21.2—27.6)	58.7 (54.5—62.9)	0.8369
Female	17.6 (14.1—21.1)	24.2 (22.5—25.9)	58.2 (54.7—61.8)	
*Age Group*				
20–29	40.4 (32.0—48.7)	37.4 (32.8—42.1)	22.2 (16.9—27.6)	< 0.0001
30–39	28.8 (23.8—33.9)	30.4 (26.5—34.3)	40.8 (36.4—45.2)	
40–49	17.1 (13.5—20.7)	30.8 (25.3—36.3)	52.2 (45.5—58.9)	
50–59	7.4 (3.2—11.6)	22.6 (16.8—28.4)	70.0 (64.4—75.5)	
60–69	2.8 (1.0—4.5)	12.9 (10.1—15.7)	84.3 (81.1—87.6)	
70–79	1.2 (0.0 – 3.0)	4.7 (2.3—7.2)	94.1 (91.1—97.1)	
80–89	1.4 (0.4—2.4)	8.8 (6.1—11.5)	89.8 (87.5—92.1)	
*Race*				
Hispanic	19.7 (16.0—23.3)	28.3 (25.0—31.6)	52.0 (47.8—56.3)	< 0.0001
Non-Hispanic White	16.5 (13.2—19.8)	22.3 (19.3—25.3)	61.2 (56.5—66.0)	
Non-Hispanic Black	16.6 (13.6—19.6)	26.0 (24.2—27.7)	57.4 (54.7—60.2)	
Asian	25.7 (20.7—30.7)	33.0 (28.7—37.4)	41.3 (37.67—44.9)	
Other or multiple races	10.7 (4.4—17.0)	22.4 (12.8—32.0)	66.9 (55.5—78.3)	
*Education Level*				
Not High School Graduate	16.0 (11.3—20.6)	22.6 (18.1—27.1)	61.5 (55.3—67.6)	0.3248
High school and above	17.5 (14.6—20.3)	24.5 (22.4—26.5)	58.0 (54.4—61.7)	
*Income to Poverty Ratio*				
At or below poverty level	18.4 (14.4—22.5)	24.4 (21.7—27.0)	57.2 (52.6—61.8)	0.6229
Above poverty level	16.8 (13.8—19.7)	24.2 (22.4—26.1)	59.0 (55.1—62.9)	
*Household Income*				
Below $20,000	16.9 (12.6—21.2)	22.0 (18.4—25.6)	61.1 (56.1—66.1)	0.4839
Above $20,000	17.4 (14.3—20.5)	24.3 (22.6—26.1)	58.3 (54.6—62.1)	
*Health Insurance*				
Yes	16.0 (13.4—18.5)	22.7 (20.8—24.6)	61.4 (58.2—64.5)	< 0.0001
No	25.6 (19.4—31.8)	34.0 (28.5—39.6)	40.4 (34.0—46.8)	
*Have regular place for healthcare*				
Yes	14.5 (12.0—17.0)	22.2 (20.4—24.0)	63.3 (60.0—66.7)	< 0.0001
There is no place	30.0 (24.3—35.7)	33.7 (29.5—37.9)	36.3 (31.6—41.0)	
There is more than one place	8.3 (0.0—23.7)	19.3 (1.7—37.0)	72.4 (53.5—91.3)	

When using a household income cutpoint of USD 20,000, the associated multimorbidity OR was 0.88 (95% CI 0.67 to 1.15, *p*-value = 0.33). As the income cutpoint increased, the ORs changed to (USD 35, 000: OR = 1.02, 95% CI 0.76 to 1.36, *p* = 0.91), (USD 45,000: OR = 1.08, 95% CI 0.76 to 1.54, *p* = 0.63), (USD 55,000: OR = 0.99, 95% CI 0.72 to 1.37, *p* = 0.97), (USD 65,000: OR = 1.16, 95% CI 0.84 to 1.62, *p* = 0.34). There is therefore the suggestion of a linear shift towards greater multimorbidity in the lower income group as income range widened, but these effects were always statistically non-significant.

In our primary regression, we stratified education level into a binary indicator (not graduated from high school /high school graduation and above). When using a 2-level education stratification, there was no effect of education on multimorbidity (OR = 0.91, 95% CI 0.58 to 1.43, *p* = 0.67). To explore the importance of this cutpoint, we chose a finer education stratification – using a 5-level categorical variable (less than 9^th^ grade, 9-11^th^ grade, high school graduate, some college, college graduate or above). This had little effect on the statistical importance of education on our definition of multimorbidity. There is some suggestion of increasing multimorbidity among those with more education, with the exception of college graduates.

**Fig. 1:**
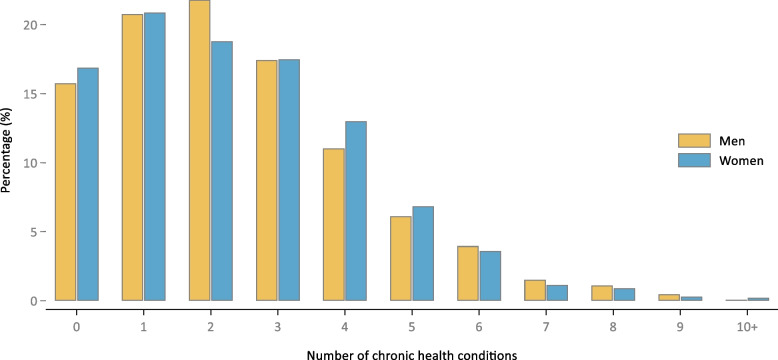
Prevalence of multimorbidity by number of chronic conditions among US adults: NHANES, 2017–18

Figure 1 is a histogram of the prevalence of multimorbidity among US adults by number of conditions considered in this study. There was no significant difference in the distribution between men and women. A total of 23.1% of the population had 2 chronic conditions, 15.3% had 3 conditions, 9.7% had 4 conditions, and 10.5% had 5 or more chronic conditions.

Table 3 lists the prevalence of pairs of comorbid chronic health conditions in US adults by sex. Hyperlipidemia, obesity, hypertension, arthritis, and diabetes were found to be the leading comorbid conditions among both men and women.

**Table 3:**
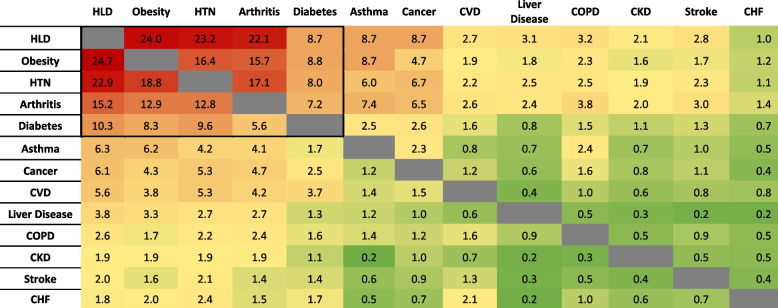
Prevalence (%) of pairs of comorbid chronic health conditions, by sex in US adults > 20 years old. NHANES, 2017–18

The proportions above and to the right of the gray diagonal are for women, and the proportions below and to the left of the gray diagonal are for men

*HLD* Hyperlipidemia, *HTN* Hypertension, *CVD* Cardiovascular disease, *COPD* Chronic obstructive pulmonary disease, *CKD* Chronic kidney disease, *CHF* Chronic heart failure

Table 4 presents our primary regression result, and represents our identified predictors of overall multimorbidity, using our chosen complement of thirteen multimorbidity components.

**Table 4:** Logistic Regression Analyses of Factors associated with Multimorbidity in US adults: NHANES, 2017–18

Sociodemographic Characteristic	Odds Ratio Point Estimate	95% Wald Confidence Limits	*P*-value from Pairwise Least Square Means
Gender: Female vs Male	0.87	0.72 - 1.04	0.132
Age Range 20–29 vs 80–89	0.04	0.03 - 0.07	< 0.0001
Age Range 30–39 vs 80–89	0.11	0.07 - 0.16	< 0.0001
Age Range 40–49 vs 80–89	0.20	0.12 - 0.32	< 0.0001
Age Range 50–59 vs 80–89	0.36	0.21 - 0.61	< 0.0001
Age Range 60–69 vs 80–89	0.84	0.52 - 1.36	0.486
Age Range 70–79 vs 80–89	1.94	0.97 - 3.89	0.062
Race Hispanic vs non-Hispanic White	1.11	0.85 - 1.47	0.437
Race Asian vs non-Hispanic White	0.44	0.35 - 0.57	< 0.0001
Race Non-Hispanic Black vs non-Hispanic White	1.13	0.95 - 1.35	0.165
Race Other or multiple races vs non-Hispanic White	1.31	0.71 - 2.41	0.391
Education High school and Above vs Not High School Graduate	0.79	0.54 - 1.16	0.223
Household Income Less than $20,000 vs More than $20,000	0.91	0.67 - 1.24	0.555
Income/Poverty Ratio Above Poverty vs At or Below Poverty	0.64	0.46 - 0.91	0.013
Has Insurance No vs Yes	0.63	0.40 - 1.01	0.053
Has Place of Healthcare: There is no place vs Yes	0.61	0.42 - 0.88	0.008
Has Place of Healthcare: There is more than one place vs Yes	1.75	0.68 - 4.49	0.247

Logistic regression showed a statistically significant lower likelihood of multimorbidity for those above compared to those at or below the poverty level; OR 0.64 (95% CI 0.46—0.88, *p* = 0.013). Multimorbidity was also demonstrated a statistically borderline association with having health insurance (*p* = 0.05). However, a lack of regular access to care was associated with a decreased likelihood of multimorbidity (OR 0.61 (95% CI 0.42 – 0.88, *p* = 0.008). Younger age (relative to age group 80–89 years) was also associated with a decreased likelihood of multimorbidity, as was Asian race (OR 0.40; 0.35 – 0.57; *p* < 0.001).

**Fig. 2:**
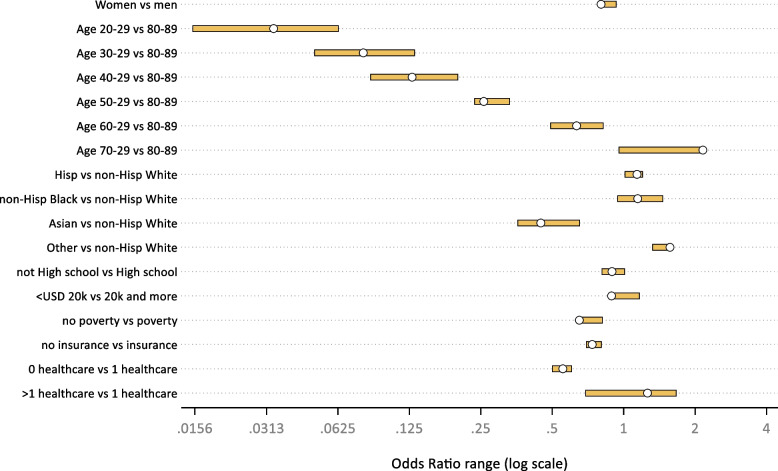
Odds Ratio range for each model predictor when removing one component at a time from the multimorbidity definition. White circle represents the observed odds ratio from the primary regression (article, Table 4)

**Fig. 3:**
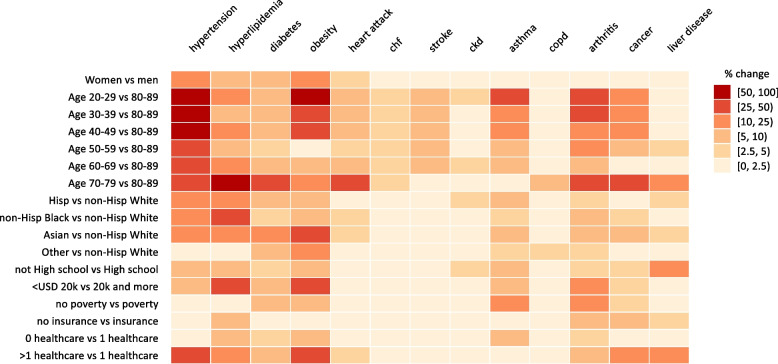
Heatmap showing the percentage change in odds ratio size when removing one component at a time from the multimorbidity definition, compared to the primary regression OR (article, Table 4)

We next performed a sensitivity analysis of our regression removing one component of the multimorbidity definition at a time and re-running the regression model, creating 13 additional sets of odds ratios. From this analysis, we plotted the range of Odds Ratios for each predictor (Fig. 2) and calculated the odds ratio percentage change for each sensitivity-based OR compared to the primary OR. We next plotted these percentage changes as heat maps to visualize the importance of each multimorbidity component on our regression predictors and Fig. 3 shows the percentage change.

Based on the OR ranges associated with each model predictor presented in Fig. 2, several age groups (20–29, 30–39, 40–49, and 70–79), along with access to places for healthcare, were most susceptible to changes in our multimorbidity definition, and were therefore associated with the widest OR ranges.

Based on the associated heatmap (Fig. 3), the removal of hypertension, hyperlipidemia, obesity and to a lesser extent asthma, arthritis and cancer individually from the model, had the greatest effect on several model predictors, causing effect size changes in excess of 25 percent for at least one model predictor.

## Discussion

Multimorbidity is highly prevalent among the adult US population, and this has remained relatively stable over the last 10 years [9]. The prevalence of multimorbidity was found to increase steadily among age groups across the lifespan and is known to be associated with higher levels of disability, immobility, loss of independence, and mortality among the elderly [1–4, 15]. A significant proportion of the young adult population was also found to suffer from multimorbidity, largely attributable to obesity and hyperlipidemia, which places them at high risk for poor cardiovascular health outcomes in later life, especially for those whose chronic conditions started during childhood [16]. Although the US Preventive Services Task Force has found no evidence to support universal screening for dyslipidemia in children [17], it has found that primary care interventions are effective for weight management in children and adolescents [18]. Public health policies should be aimed at reducing the prevalence of obesity among children and adolescents, and thus reducing the prevalence of multimorbidity among young adults [19]. Of note, in this study, logistic regression analysis indicated that Asians were at lower risk of multimorbidity.

Covariate-adjusted survival analysis has shown that among the older adult population in the US the "complex cardiometabolic" class of diseases leads to the highest mortality [20], and this study confirmed the steady increase in prevalence of this class of diseases across the lifespan leading to a high prevalence among the elderly. Three to six percent of the population studied, and about 10% of those above age 50 were found to have all four risk factors for cardiovascular disease and stroke, namely, hypertension, obesity, hyperlipidemia, and diabetes.

Of relevance to the ongoing pandemic, after age, obesity has been found to be the second most significant risk factor for severe disease and death from COVID-19 [21]. In this study it was found that the prevalence of obesity is about 40% for all racial/ethnic groups, but slightly higher for Blacks. The high prevalence of existing multimorbidity in the US population also contributed to the large adverse health impact of COVID-19, possibly linked to high levels of background inflammation [22]. This association was linked to race [23], such that Black and American-Indian/Alaskan Natives had the highest levels of mortality among younger age groups (< 65 years), and Black and Hispanics among the older age groups (≥ 65 years) [24].

The subsequently reported high incidence of severe disease and death from COVID-19 seen in older Americans is consistent with the high prevalence of multimorbidity reported in this study. Although race or ethnicity was not associated with increased risk of multimorbidity, obesity and hypertension were found to be more prevalent among Blacks and Hispanics (Table 1). Blacks and Hispanics are also particularly disadvantaged in many social determinants of and are more likely to work as frontline workers [25], where they have been at greater risk of contracting COVID-19 infection.

It is important to note that the prevalence of multimorbidity was found to be higher among those with regular access to healthcare or having more than one place where they obtained care, likely due to a higher chance of being diagnosed with chronic illnesses. The dataset, however, did not provide detailed information about when or where diagnoses were made or the duration of illness. Logistic regression analyses (Table 4) confirmed that persons who did not have a place for healthcare had statistically lower rates of multimorbidity. This relationship may seem counterintuitive, but it is likely due to lack of diagnosis, such that people were unaware of having multiple chronic conditions. Disparities in access to healthcare played a role in differential death rates among ethnic minorities and residents of rural areas and areas with a scarcity of primary care physicians [26–28]. Underdiagnosis and lack of management of multimorbid chronic conditions likely played a role in those disparities. Therefore, the higher rates of morbidity and mortality from COVID-19 among these groups is a multifactorial phenomenon and will require further study to evaluate the interplay of having multimorbid conditions and lower access to care.

The present study did not find associations between multimorbidity or obesity with education level less than high school versus high school and above, nor with income level below or above $20,000, likely because of the uniformly high levels of obesity among the entire population. One MMWR report based on NHANES 2011–2014 did find an association for obesity with age-adjusted education and income levels, but not consistently for different racial groups [29]. In the present study income-to-poverty ratio below poverty or above poverty levels did show a significant difference in the odds ratio for multimorbidity (Table 2). Poverty has been causally associated with poor outcomes from other infectious diseases [30] and there is now clear evidence that poverty also led to worse outcomes from COVID-19 [31, 32]. The association between poverty and multimorbidity is one possible mechanism for the impact of poverty on infectious diseases in general and COVID-19 in particular [33].

### Implications for policy, practice and future research

This analysis adds to the growing literature documenting that more than half of the adult population in the USA lives with more than one noncommunicable disease/condition; that obesity, hyperlipidemia and hypertension are the main co-morbid conditions; that older adults (70–79 years of age) are more likely to have more than one noncommunicable disease/condition; and that access to health care has a significant impact on health outcomes for people with noncommunicable diseases. This analysis also revealed that young adults, aged 20–29 years of age are an important group for multi-morbidity care, as almost a quarter of this population has two or more noncommunicable disease/condition, notably obesity and hyperlipidemia.

Our findings point to the need to alter perspectives in health policies and services, that tend to focus on individual diseases rather than a comprehensive people-centered perspective, to ensure integrated care for people with noncommunicable diseases. It also points to the need for health policies to improve health insurance coverage, ensuring access and quality of primary care for the range of co-morbid noncommunicable diseases/conditions that are most prevalent in the USA, adapting services to the needs of young adults as well as older adults.

Most importantly, this analysis reveals the failures in NCD primary prevention, especially among young adults, given the large proportion of multi-morbid conditions in those 20–29 years of age. This speaks to the need to improve public health prevention policies, to promote healthy eating, physical activity, obesity prevention and reduction of hyperlipidemia.

Future research would be valuable on the prevalence and determinants of multi-morbid conditions, by age group, ethnicity, and other socio-economic factors in order to continue documenting the situation of noncommunicable diseases/conditions in the USA. Future research would also be beneficial on the impact of health policies, for example improvements in health insurance coverage or access to preventive care services, on the situation of multiple noncommunicable diseases/conditions.

## Conclusions

High levels of multimorbidity with obesity, hyperlipidemia, hypertension, and diabetes among US adults likely exacerbated the high levels of severe disease and death from COVID-19 during the early period of the pandemic. While associations between chronic conditions, particularly NCDs and COVID-19 are known, the true impact of multimorbid conditions on complications and death due to Covid-19 continues to be largely under-recognized. This will be critical to strengthen public health policies aimed at prevention, early detection, and optimal management of comorbid conditions in vulnerable populations with poor social determinants of health. Although race and ethnicity were not found to be significant determinants of increased likelihood of multimorbidity in this study, analysis of individual cardiometabolic diseases showed significant differences among different ethnic groups. Addressing the socioeconomic factors behind these disparities and gaps in care will be key to improving the overall health of all people living in the U.S., such that the country will more appropriately address the ongoing challenge of the pandemic and be well prepared for future public health emergencies. Doing so requires policies and interventions to address the three main factors highlighted in the current study as being impactful on the health of the US adult population: obesity, poverty, and lack of regular access to quality healthcare. Increasing public health policies to promote a healthy diet and physical activity, as well as Medicare coverage and other measures to promote access to healthcare are key. Comprehensive social and public health policy approaches are needed to reduce disparities in the social determinants of health. These policies could include improving access to healthy foods for school children and those living in socioeconomically deprived circumstances, as well as policies aimed at poverty alleviation [34], and providing universal access to healthcare for all [35]. Implementation of these policies will require collaboration between federal and state governments, public health institutions, and the healthcare and insurance industries [36, 37]. Ultimately, they will improve the general health and welfare of all people living in the U.S.

## Data Availability

The analyses were based on de-identified data. The datasets analyzed during the current study are available in the CDC/National Center for Health Statistics repository, https://wwwn.cdc.gov/nchs/nhanes/continuousnhanes/default.aspx?BeginYear=2017
